# Impact of Preparation Thickness and Restorative Material on the Marginal Fit of CAD‐CAM Complete Crowns

**DOI:** 10.1155/ijod/3935712

**Published:** 2026-07-14

**Authors:** Éric Arnold dos Santos Brito, Thayara Coelho Metzker, Ingrid Bandeira Sanches, Adriana Oliveira Carvalho, Emilena Maria Castor Xisto Lima

**Affiliations:** ^1^ Postgraduate Program in Oral Rehabilitation at the School of Dentistry of Ribeirão Preto, University of São Paulo (FORP-USP), Ribeirão Preto, São Paulo, Brazil; ^2^ Department of Dentistry, Bahiana School of Medicine and Public Health (EBMSP), Salvador, Bahia, Brazil, bahiana.edu.br; ^3^ Department of Dentistry, Feira de Santana State University (UEFS), Feira de Santana, Bahia, Brazil, uefs.br; ^4^ Department of Dental Clinic, University of Bahia (FOUFBA), Salvador, Bahia, Brazil

## Abstract

**Statement of Problem:**

A lack of consensus exists on whether the preparation thickness and choice of restorative material can affect the marginal adaptation of complete CAD‐CAM crowns.

**Purpose:**

To evaluate the impact of preparation thickness and restorative material on the marginal adaptation of CAD‐CAM complete crowns.

**Materials and Methods:**

Two human mandibular right second molars were prepared with the following characteristics: conventional preparation (CP)—1.0 mm chamfer, 2 mm reduction of the occlusal surface, ~6° convergence angle, and 1.5 mm axial reduction; ultrathin preparation (UFP)—0.5 mm finish line, 0.7 mm reduction of the occlusal and axial surface. The teeth were scanned using an intraoral scanner (IOs), and the crowns were designed and milled. Ten crowns were produced in each group (*n* = 10): feldspathic ceramic (FC), resin composite (RC), lithium silicate ceramic reinforced with zirconia (LSZ), and lithium disilicate ceramic (LDS). The marginal gap was analyzed at 4 points using a stereomicroscope. Data analysis used two‐way analysis of variance (ANOVA) and the Tukey test (*α* = 0.05).

**Results:**

CP (65.00 ± 16.62 to 84.22 ± 20.87 µm) showed the lowest marginal discrepancy values, with a statistical difference from UFP (110.40 ± 28.64 to 183.83 ± 40.23 µm). In the CP group, there was no statistically significant difference between the materials. In the UFP, there was a difference between FC and RC compared to LSZ and LDS.

**Conclusions:**

The preparation’s thickness impacted the marginal adaptation of CAD‐CAM full crowns, and the CP showed a lower discrepancy compared to the ultrathin preparation. The performance of the materials in the CP was similar. However, there was better marginal adaptation of FC crowns and RCs in ultrathin preparations.

## 1. Introduction

### 1.1. Clinical Implications

Marginal adaptation is a crucial condition that impacts the clinical performance and longevity of restorations. Despite different types of preparations and materials acting as influencing factors in the marginal adaptation of CAD‐CAM full‐crown restorations, the results of this study were acceptable.

Metal‐free indirect restorations can resemble dental structures with better translucency and color, making them more esthetically favorable. The preparation of metal–ceramic crowns requires more significant tooth structure wear than the preparation of all‐ceramic crowns [1]. The development of adhesive approaches allows the use of indirect restorations with minimal intervention, resulting in less reduction of the tooth structure [1, 2]. Thus, with the principles of minimally invasive dentistry, the importance of conserving dental tissue in the preparation of full crowns has become paramount, leading to the emergence of ultrathin preparations (UFPs). Improvements in adhesion and restorative materials are essential for this process [3, 4].

Ultraconservative indirect preparations began with veneers and progressed to partial restorations, such as onlays and full crowns [1, 5, 6]. However, although there may be a trend towards minimally invasive preparations, it is still unclear what restrictions can be imposed regarding the use of materials and fabrication methods, which can lead to an increase in restoration failures.

Improvements in ceramics and resins offer new applications for their use. Machinable versions of these materials have been introduced into CAD‐CAM systems [7]. Through scanning, it is possible to visualize preparations on a screen with magnification, enabling the detection of areas that require adjustments [7], and the associated milling process has some advantages, such as a higher degree of precision and shorter fabrication time, which can be carried out in a single session [8]. Through this technology, it is possible to achieve crowns with a higher degree of precision in marginal adaptation. Studies have demonstrated better marginal adaptation of prosthetic pieces manufactured using the CAD‐CAM system compared to those with conventional techniques [9, 10]. The fundamental requirements for restorative materials used for the fabrication of CAD‐CAM crowns are good machinability, fracture resistance, and minimal finishing procedures [4]. In this context, improvements in the quality of materials, scanners, software, and milling machines have made it possible to produce ultrathin indirect restorations [1].

Ceramic systems have been widely studied due to their esthetics, biocompatibility, low thermal conductivity, reduced wear rate, high degree of polishability, and fracture resistance [6]. Another material widely used for crown fabrication is resin composite (RC) blocks, which consist of fine ceramic particles immersed in a polymeric matrix [7]. These possess excellent microstructural properties and a high degree of conversion. Magne et al. [1], Dauti et al. [7], and de Paula Silveira et al. [8] reported that the fabrication of crowns with RCs has some advantages over pure ceramic crowns: less wear on the drills and reduced milling time, no need for firing, better polishing, ease of adjustment, low abrasiveness to the opposite tooth, continuous and well‐fitting margins after milling, and a lower modulus of elasticity than that of ceramics, which allows for stress absorption through deformation, making this material ideal for minimally invasive restorations.

Restorative success is analyzed through various elements, including mechanical requirements, esthetics, and marginal adaptation [4]. The marginal gap is the distance between the edge of the finishing line of the prepared tooth and the cervical margin of the restoration [11, 12]. The clinically acceptable marginal discrepancy threshold [3] is up to 120 µm for crowns’ conventional method and for CAD‐CAM 50 and 100 µm [11, 13]. Values higher than these are considered marginal misfits and can lead to biological complications (biofilm accumulation, microleakage, caries, and pulpal and periodontal inflammation) and mechanical issues (reduced fracture resistance and retention loss) [11, 14–16].

This in vitro study aimed to assess the influence of preparation thickness and restorative material on the marginal adaptation of CAD‐CAM complete crowns. The null hypotheses were that no significant difference would be found among the marginal adaptation of the four materials tested and that the marginal adaptation of these restorations would not be influenced by the thickness of the preparation (conventional and ultrathin).

## 2. Material and Methods

The present study was approved by the Research Ethics Committee of the Faculty of Dentistry of the Federal University of Bahia (Protocol Number: 94713718.0.1001.5024). The study design consisted of the selection of two extracted human mandibular molars without caries. The specimens were cleaned to remove surface debris and stored in a 0.01% thymol solution in order to inhibit microbial growth. The specimens were maintained in a humid environment using a sealed metal container with a moistened sponge to prevent dehydration.

The mandibular right second molars were mounted together with adjacent teeth in typodont models (Pronew) and prepared for full‐coverage ceramic restorations using a chamfer‐finish line. Two preparation designs were established. For the conventional preparation (CP), a 2 mm reduction was performed on the occlusal surface, with ~1.5 mm axial reduction and a convergence angle of about 6°. The finish line was positioned above the cemento–enamel junction and consisted of a circumferential chamfer measuring 1.0 mm. For the UFP, both axial and occlusal reductions were standardized at 0.7 mm, maintaining a convergence angle of ~6°, with the finishing line also located above the cemento–enamel junction and defined as a 0.5 mm chamfer.

Tooth preparations were performed using diamond rotary instruments mounted on a multiplier contra‐angle handpiece (T3‐Line; Sirona Dental Systems GmbH). The preparation sequence consisted of FG 3216 (KG Sorensen) for the creation and connection of orientation grooves, FG 3203 (KG Sorensen) for the removal of the proximal contact point, FG 3216 for chamfer formation, and FG 4138 (KG Sorensen) for finishing.

### 2.1. Intraoral Scanning

For digital acquisition, 10 intraoral scans were obtained for each preparation group using a CEREC Omnicam scanner (Sirona Dental Systems GmbH). The digital models were processed using CAD software (CEREC inLab SW 4.5; Sirona Dental Systems GmbH) for restoration design. The operator‐defined design parameters were as follows: radial and occlusal spacer of 80 μm; proximal and occlusal contact resistance of −25 μm; dynamic contact force of −25 μm; minimum radial thickness of 700 μm; minimum occlusal thickness of 900 μm; and marginal thickness of 80 μm (Table 1). All crowns were designed with a standardized occlusal anatomy and equal occlusogingival height.

**Table 1:** Parameters used for crown fabrication.

Conventional preparation	Ultrathin preparation
Radial and occlusal clearance: 80 μm	Radial and occlusal clearance: 80 μm
Proximal contact resistance, occlusal contact resistance, and dynamic contact force: −25 μm	Proximal contact resistance, occlusal contact resistance, and dynamic contact force: −25 μm
Minimum radial thickness: 1000 μm	Minimum radial thickness: 700 μm
Minimum occlusal thickness: 1000 μm	Minimum occlusal thickness: 900 μm
Margin thickness: 100 μm	Margin thickness: 80 μm
Ramp angle: 60 μm	Ramp angle: 60 μm

After the computer‐aided design stage, the files were transferred to a milling unit (CEREC inLab MCXL; Sirona Dental Systems GmbH). All scanning and design procedures were performed by a single operator. For each digital design, four crowns were milled, corresponding to the different restorative materials. Milling burs were replaced after every five specimens in each group.

Ten crowns were fabricated for each group (*n* = 10), considering the combination of preparation type and material: feldspathic ceramic (FC), RC, zirconia‐reinforced lithium silicate ceramic (LSZ), and lithium disilicate ceramic (LDS). The sample size was defined based on previous in vitro studies with a similar methodology [17, 18]. The materials used are given in Table 2. Lithium disilicate crowns were subjected to crystallization firing in a ceramic furnace (Programat CS2; Ivoclar AG) according to the manufacturer’s instructions.

**Table 2:** The materials involved in the study.

Brand name	Composition	Company	Reference/batch
Cerec Blocs	Feldspathic ceramic	Sirona (Bad Sackingen, Bensheim, Germany)	6484526/54001
IPS e.max ‐ CAD	Vitroceramic of lithium disilicate	Ivoclar ‐ Vivadent (Schaan, Liechtenstein)	626409/X20078
Celtra Duo	Lithium silicate glass–ceramic reinforced with zirconia	Dentsply Sirona (Hanau, Wolfgang, Germany)	5365411225/1600 0912
Grandio Blocs	Resin composite	VOCO (Cuxhaven, Germany)	6022/1731082

For marginal discrepancy analysis, each crown was positioned on its respective prepared tooth and stabilized using a C‐shaped clamp to ensure consistent positioning during stereomicroscopic evaluation. Measurements were performed using a stereomicroscope (Optima MDCE‐5ª 2.0; Hiperquímica) at 45× magnification, evaluating the buccal, lingual, mesial, and distal surfaces. The captured images were exported to CorelDraw X7 software for analysis.

Marginal adaptation was evaluated by measuring the vertical discrepancy between the crown margin and the preparation finish line. Four reference points were defined at the midpoint of each surface (buccal, lingual, mesial, and distal). At each location, three measurements were obtained along the long axis of the tooth, resulting in 12 measurements per specimen. The mean value of these measurements was used for statistical analysis. All measurements were performed by a single calibrated examiner [19, 20].

### 2.2. Statistical Analysis

It began with exploratory procedures to assess the homogeneity of variances and data distribution. The Levene test was used to verify the homogeneity. Subsequently, a two‐way analysis of variance (ANOVA) was performed, followed by Tukey’s post hoc test. All analyses were conducted using SPSS Statistics software (version 19.0; IBM Corp.), with the level of significance set at α = 0.05.

## 3. Results

Statistical analysis showed significance between preparations and materials (*p* < 0.001), demonstrating the dependence of one factor on the outcome of the other (Table 3). Different capital letters denote statistically relevant variations among the materials in each preparation category. Lowercase letters represent the differences between preparations for each material (Two‐way ANOVA, Tukey, *p* < 0.05). Statistically significant differences were found between the CP and UFP for all materials, with higher values of marginal discrepancy observed in crowns fabricated using the UFPs.

**Table 3:** Interaction between materials and preparations.

Interactions	Mean square	Significance
Preparations	104,674.373	*p* < 0.001
Materials	6060.348	*p* < 0.001
Materials and preparations	6222.662	*p* < 0.001

Regarding the materials tested separately for each type of preparation, it was observed that in the CP group, there were no statistically significant differences among the materials. In the UFP group, the lowest marginal discrepancy values were found in the FC (110.40 ± 28.64 µm) and RC (123.51 ± 32.44 µm) restorations, which did not differ significantly from each other. However, these values showed statistically significant differences when compared to those presented by LDS (183.83 ± 40.23 µm) and LSZ (162.54 ± 29.23 µm), which also did not differ significantly from each other (Table 4).

**Table 4:** Means and standard deviation of marginal discrepancy (µm) of complete crowns, with two types of preparation (conventional and ultrathin).

Materials	Conventional preparation (PC)	Ultrathin preparation (PUF)
Feldspathic ceramic (FC)	73.25 ± 18.19^Aa^	110.40 ± 28.64^Ab^
Resin composite (RC)	68.42 ± 11.32^Aa^	123.51 ± 32.44^Ab^
Silicate lithium reinforced with zircônia (SLZ)	65.00 ± 16.62^Aa^	183.83 ± 40.23^Bb^
Lithium disilicate (LDS)	84.22 ± 20.87^Aa^	162.54 ± 29.23^Bb^

*Note:* Distinct uppercase letters represent significant differences among the materials in each type of preparation. Lowercase letters represent the differences between preparations for each material.

## 4. Discussion

The development of materials and techniques, coupled with improvements in adhesive approaches, has made indirect restorations more durable and conservative [1]. Values ranging from 10 to 220 µm have been reported, although there is no consensus on the clinically acceptable marginal discrepancy [21, 22]. While for restorations fabricated through a digital workflow, marginal gap values between 50 and 100 µm have been described [11, 13, 23].

Although multiple finishing line designs have been proposed for indirect posterior restorations (IPR), a universally accepted classification is still lacking, and objective selection criteria remain limited. The choice of margin configuration and location should rely on careful anatomical evaluation of posterior teeth since the finishing line directly influences the available bonding surface and the extent of dentin exposure. Among the available designs, the *butt joint* margin is widely recommended for adhesive preparations because of its conservative nature, providing a flat surface with internal convergence and preserving dental tissue; it is particularly indicated in cases of severe wear requiring a vertical dimension increase and in teeth with unsupported cusps. Variations such as the inclined chamfer or inclined bevel introduce an ~45° marginal inclination, creating a smoother tooth‐restoration transition and potentially enhancing manufacturing integration, especially in esthetically demanding situations. The shoulder finish line, generally prepared with a rounded internal angle, increases marginal material thickness and may improve rotational resistance, whereas the chamfer margin is considered more conservative for full‐coverage restorations and has been associated with improved postcementation fit. Despite these proposed advantages, evidence clarifying the effect of margin design on IPR performance remains scarce, and clinical decisions continue to depend largely on practitioner judgment [24].

This study investigated the influence of preparation thickness and restorative material on the marginal fit of full‐contour CAD‐CAM crowns. Two null hypotheses were tested: that there would be no statistically significant differences in marginal fit among the four restorative materials evaluated and that marginal fit would not be affected by preparation thickness (conventional versus ultrathin). The first hypothesis was partially rejected as no significant differences were observed among the materials when CPs were used; however, variations in marginal fit were noted with UFPs. The second hypothesis was fully rejected since statistically significant differences were found between conventional and UFPs across all tested materials.

In this study, the CP group presented marginal gap values that were considered clinically acceptable, remaining within the 120 µm threshold defined by McLean and von Fraunhofer [3] for crowns fabricated by the conventional method. However, in the UFP group, only FCs presented values within this acceptable threshold, whereas the other materials exhibited higher values. Although the results showed values above the threshold for CAD‐CAM crowns (50–100 µm) in the UFP group, discrepancy values up to 150 µm were well accepted without any clinical impairment [11, 13].

de Paula Silveira et al. [8] assessed the internal and marginal fit of complete CAD‐CAM crowns fabricated using a RC and lithium disilicate‐reinforced ceramic using two types of intraoral scanners (IOs). Regarding absolute marginal adaptation, no significant statistical variations were identified among the materials analyzed, reinforcing the findings of the present investigation. Although the composite used was Lava Ultimate and the finish line had a rounded shoulder, these factors did not affect the results. However, Tabata et al. [25] evaluated internal and marginal adaptation of crowns generated via CAD‐CAM technology and varying the material type and internal spacing and observed that at a spacing of 80 μm, the LDS materials—IPS e.max CAD (161 ± 47 μm) and RC—Lava Ultimate (148 ± 47 μm) showed a statistically significant difference between them. Kapler et al. [19] assessed the marginal fit of crowns produced using the CAD‐CAM technology with different material types. A significant difference was observed between lithium disilicate (84.22 ± 20.86 μm) and the RC Brava Block (60.95 ± 13.64 μm). However, no significant difference was found when compared to the RC Grandio Blocs (68.42 ± 11.31 μm), which is the same material used for the crowns produced via the CAD‐CAM technology in the present study. All tested materials exhibited marginal discrepancies that remained within the clinically acceptable threshold of 120 µm as proposed by McLean and von Fraunhofer [3].

The most commonly used preparations for ceramic crowns are rounded shoulders and chamfers. Akbar et al. [26] assessed the marginal fit of CEREC 3 CAD‐CAM composite crowns fabricated with two distinct finish line designs: shoulder and chamfer. The marginal discrepancies found were 65.9 ± 38.7 µm and 46 ± 9.2 µm, respectively; however, no significant statistical differences were detected among the groups. Regardless of the type of preparation used, the mean marginal discrepancy in the restorations was clinically acceptable. The values identified for the chamfer‐finish design were consistent with those of the CP group, reinforcing the results of this study. This study revealed a statistically significant difference in the marginal discrepancy values observed in the CP group (65 and 84.22 µm) compared to the UFP group (110.40 and 183.83 µm) for all tested materials. However, there is a scarcity of studies evaluating marginal adaptation in ultrathin complete crowns. According to Magne et al. [1] and Cortellini and Canale [6], ultrathin complete crowns have adequate fatigue resistance and can be considered an alternative to conservative treatment with full crowns. Cortellini and Canale [6] conducted a longitudinal investigation over 4 years to examine the clinical performance of ultrathin complete crowns, characterized by a 0.5 mm axial reduction and a 0.3 mm finish line, manufactured through both CAD‐CAM and traditional methods and concluded that there was no statistically meaningful variation between the two manufacturing techniques, the long‐term tissue response was gingival stability, and no signs of inflammation or biological complications, including secondary caries, were detected. Magne et al. [1] carried out a study assessing the survival rate and failure mode of ultrathin complete crowns fabricated using CAD‐CAM systems and observed defects at the cervical margin of milled crowns, suggesting that, in UFPs, there is a tendency for marginal misfit.

The surface finish after preparation is a crucial step in the internal and marginal fit of restorations. Tamam et al. [27] assessed tooth preparations subjected to various surface finishing protocols in monolithic FC crowns: extra‐thick (181 μm), fine (40 μm), very fine (20 μm), and very fine plus rubber polishing. The lowest marginal gap values were found in the very fine group, resulting in an improved marginal fit for feldspathic restorations within clinically acceptable limits.

Esthetic restorative materials have undergone significant development for posterior applications, driven by improvements in mechanical performance and adhesive systems. Indirect restorations, including those fabricated through CAD/CAM workflows, undergo additional polymerization under controlled conditions, resulting in a higher degree of conversion, enhanced mechanical properties, and reduced residual monomer release. Randomized clinical trials demonstrate that posterior resin restorations, whether direct or indirect, exhibit comparable longevity across 1–3, 4–7, and 8–11‐year follow‐up periods, with no statistically significant differences between techniques. Survival outcomes were not influenced by the manufacturing or cementation approach, including CAD/CAM. The predominant failure modes were fracture, secondary caries, and debonding, with secondary caries associated with polymerization stress at the tooth‐restoration interface [28].

When comparing the materials in the UMP group, the smallest marginal discrepancy values were found for the FC and RCs, which did not differ from each other. The FC used in this study was Cere Blocs, which is indicated for crowns, veneers, and onlays, allowing for milling margins up to 0.4 mm [29]. For the UFP, milling was performed with a cervical margin thickness of 0.5 mm, and the marginal discrepancy values obtained were 110.40 ± 28.64 µm. This UFP material presented a marginal discrepancy closest to the clinically acceptable limit for CAD‐CAM crowns as proposed by Euán et al. [11] and Ural et al. [13]. This can be explained by the malleability of the material owing to its higher glass portion. In addition, the crystals had a rounded shape, which facilitated milling. Like resins, FCs have a lower milling resistance than zirconia and lithium disilicate [15, 30]. According to Magne et al. [1], RCs have a low modulus of elasticity, which allows for stress absorption through deformation, making this material ideal for minimally invasive restorations. The RC used in this study was Grandio blocks, which have an inorganic filler content of 86%, excellent flexural strength, and abrasion resistance and are indicated for veneers and crowns, allowing for milling margins of up to 0.4 mm [31, 32]. Moreover, it was observed that the milled RNC crowns demonstrated superior marginal fit compared to the ceramic crowns, which tended to exhibit minor marginal imperfections [1].

Lithium silicate reinforced with zirconia is a material that has recently been available in the market. This material contains lithium oxide, silicon dioxide, and ~10% zirconia dioxide in its composition, and still, very few studies are available on its performance [21]. Atlas et al. [21] assessed the marginal adaptation of CAD‐CAM crowns composed of lithium silicate reinforced with zirconia, fabricated after an initial preparation with loupes and subsequent refinement using either loupes or a microscope. The discrepancy found in the preparation with a microscope (35.6 ± 110.6 µm) was statistically significant compared to the initial preparation with loupes (145.0 ± 259.6 µm), resulting in a more precise marginal fit [21].

In this study, the lithium disilicate glass–ceramic used was IPS e.max CAD, which comprises a glass phase and two crystalline phases that provide adequate translucency [33]. Additionally, the long, interconnected crystals in the glass matrix contributed to a reduction in crack propagation. After sintering, this material has a biaxial flexural strength of 407 ± 45 MPa, an elastic modulus of 95 GPa, and a fracture toughness of ~3 MPa½, favoring satisfactory mechanical performance [33]. It has various indications, including posterior and anterior, onlays, inlays, and crown veneers. For this material, the minimum thickness of the ceramic structure was 0.4 mm [33]. Besides having a larger crystalline portion, the crystallization process inherent in the production of restorative materials can lead to contraction, favoring an increase in the marginal misfit [33].

Aboushelib et al. [34] conducted a study to evaluate the internal and marginal fit in LDS veneers with 0.7 mm preparations on the buccal surface, fabricated using the CAD‐CAM with extraoral scanning and the conventional method. The authors found that pressed ceramic veneers demonstrated a reduction in marginal discrepancies at the cervical margins compared with those of milled ceramic veneers. Veneers fabricated by the pressing method showed better marginal adaptation, greater homogeneity, a thinner cement thickness, and better resistance to microleakage than milled ceramic veneers. This may be explained by limitations in the camera hardware, difficulty in scanning owing to the preparation thickness, and milling limitations owing to the thin margin thickness [33]. These findings are in agreement with the results of the present study, in which marginal discrepancy values were higher in the UFP group despite full‐crown preparation.

Tan and Dudley [35] assessed the marginal discrepancies of lithium disilicate crowns fabricated using different milling units and suggested that the number of axes may affect the precision of the milled crown fit. However, Beuer et al. [36] suggested that precision may be more closely related to digitalization and data processing than to the number of axes. The number of burs used may be associated with greater marginal discrepancies due to the reduced cutting efficiency caused by the loss of abrasive particles. Decreased cutting ability can increase friction and stress on the ceramic block, leading to microfractures in the ceramic and marginal misfits [34]. To minimize potential biases in this investigation, all specimens were milled using the same milling unit and operated by a single technician, alternating between their respective groups. Thus, these factors influenced all groups equally.

The limitations of this study include the fact that only one natural tooth was prepared to minimize the possibility of errors and the noncementation of crowns on the prepared tooth. Other limitations include the absence of a simulation of intraoral conditions, such as saliva, moisture, patient movement, and difficulty in scanning interproximal areas.

Clinical evidence and additional research are required to assess the marginal adaptation of UFPs as this type of preparation promotes greater preservation of the dental structure.

## 5. Conclusions

From the outcomes observed in this study, the following conclusions can be established:

1. The preparation thickness influenced the marginal adaptation of CAD‐CAM complete crowns. Compared to the UFP, the CP showed a lesser marginal discrepancy.
2. The materials tested demonstrated comparable performance in CPs; however, ultrathin FC and RC crowns showed superior marginal adaptation.

## Funding

No funding was received for this manuscript.

## Conflicts of Interest

The authors declare no conflicts of interest.

## Data Availability

The data that support the findings of this study are available from the corresponding author upon reasonable request.
